# Machine learning–accelerated design and synthesis of polyelemental heterostructures

**DOI:** 10.1126/sciadv.abj5505

**Published:** 2021-12-22

**Authors:** Carolin B. Wahl, Muratahan Aykol, Jordan H. Swisher, Joseph H. Montoya, Santosh K. Suram, Chad A. Mirkin

**Affiliations:** 1 Department of Materials Science and Engineering, Northwestern University, Evanston, IL 60208, USA.; 2 International Institute for Nanotechnology, Northwestern University, Evanston, IL 60208, USA.; 3 Toyota Research Institute, Los Altos, CA 94022, USA.; 4 Department of Chemistry, Northwestern University, Evanston, IL 60208, USA.

## Abstract

In materials discovery efforts, synthetic capabilities far outpace the ability to extract meaningful data from them. To bridge this gap, machine learning methods are necessary to reduce the search space for identifying desired materials. Here, we present a machine learning–driven, closed-loop experimental process to guide the synthesis of polyelemental nanomaterials with targeted structural properties. By leveraging data from an eight-dimensional chemical space (Au-Ag-Cu-Co-Ni-Pd-Sn-Pt) as inputs, a Bayesian optimization algorithm is used to suggest previously unidentified nanoparticle compositions that target specific interfacial motifs for synthesis, results of which are iteratively shared back with the algorithm. This feedback loop resulted in successful syntheses of 18 heterojunction nanomaterials that are too complex to discover by chemical intuition alone, including extremely chemically complex biphasic nanoparticles reported to date. Platforms like the one developed here are poised to transform materials discovery across a wide swath of applications and industries.

## Teaser

Machine learning accelerates materials discovery by suggesting targets, yielding exceptionally complex biphasic nanoparticles.

## INTRODUCTION

Nanoparticles (NPs) expand the design space of functional materials from bulk to surface and interface dominated length scales where previously unknown physical and chemical properties emerge (*1*). Efforts to explore this vast space and identify previously unidentified structures, primarily through size, shape, and composition control in an attempt to elucidate structure-function relationships, have been ongoing for decades. Using both experimental combinatorial approaches (*2*–*4*) and computational methods (*5*–*9*), new NP compositions and structures have been found for applications ranging from energy storage and conversion to therapeutics (*10*–*12*). Metallic NPs, for instance, show particular promise as heterogeneous catalysts for industrially critical reactions, such as oxygen reduction and evolution, hydrogen evolution, and CO_2_ reduction, where complex design features at the nanoscale can have profound effects on catalyst performance (*1*, *13*). NP size is a widely studied design axis, as changes in surface-to-volume ratios or surface atom coordination play a vital role in controlling properties on a per-atom basis, as in catalysis of CO_2_ reduction on Au NPs (*14*). Beyond size, variation of phase morphology is emerging as another axis that can greatly expand the design space in nanostructured materials, as complex interface motifs between phases can impact properties through numerous mechanisms. For instance, catalytic activity can be enhanced by inducing lattice strain on the particle surface through a deliberately selected core (e.g., faster oxygen reduction on Pt in PtPb@Pt core-shell NPs) (*15*) or controlling the arrangement of active sites across an interface (e.g., as in tandem catalysts) (*16*). Yet, despite the potential impact interfaces can have on particle performance or properties, there are few robust and controlled methods for deliberately producing these interfaces in nanostructures.

Recent developments in the synthesis of multimetallic NPs have greatly expanded their design space (*17*), with elemental complexities reaching 10 elements in a single particle and phase motifs ranging from homogenous alloys to core-shell particles and even to heterojunction multiphasic particles with up to six unique interfaces coexisting in a single particle (*18*–*20*). Among the techniques, scanning probe block copolymer lithography (SPBCL), wherein a scanning probe deposits a miniscule volume of block copolymer and metal salts to form a volume-confined nanoreactor, has emerged as a particularly robust method for the mass synthesis of heterojunction NPs (*2*, *6*, *18*, *21*–*24*). In SPBCL, the block copolymer serves as a solvent to facilitate the aggregation and coarsening of metal precursors into single particles, resulting in spatially defined arrays of size and composition controlled NPs. Past examples include NPs with as many as seven elements and six unique interfaces and libraries of nanostructures with more than a million positionally encoded discrete nanostructures—so-called “megalibraries” (*18*). This platform, therefore, is an excellent means for systematically investigating the properties of heterojunction NPs.

However, given the vast possible combinations of composition and particle size accessible through SPBCL and megalibraries in general (e.g., >120 million pentametallic combinations when considering particles between 10 and 20 nm in 2-nm increments), particularly when generating NP megalibraries in combinatorial synthesis (*2*), experimentation alone is insufficient to realize structures of interest under finite resources and in reasonable time scales. Accurate first-principles or atomistic-level modeling of NP properties, particularly those pertaining to interfaces, typically comes at a high computational cost that renders them viable for case studies but prohibitive for navigating the vast combinatorial space of multimetallic nanostructures. Thus, despite the emergent properties and performance gains that designer heterojunctions may impart, rational control of interfaces between distinct phases in nanomaterials remains a formidable challenge.

Machine learning, particularly in the context of Bayesian optimization (BO) (*25*–*27*), can help circumvent these bottlenecks by learning a relationship between the targeted property of NPs and design parameters using past experimental results and can guide the expensive and labor-intensive synthetic search through an iterative feedback loop between physical experiments and optimization algorithm. Here, we demonstrate a highly efficient closed-loop integration of SPBCL synthesis and scanning transmission electron microscopy (STEM) imaging of multimetallic NPs with BO, focusing on accelerated discovery of isolated (single) interface, two-phase quaternary, quinary, and senary multimetallic NPs in a broader eight-dimensional (8D) element space. Our results show that the rational compositional design of heterojunctions in NPs is possible through BO when researchers “stay in the loop,” paving the way for the machine learning–guided design of more complex, functional multimetallic NPs.

## RESULTS

### Design and simulation of an optimization approach for NP discovery

The complexity and breadth of the phase morphologies NPs can adopt increases rapidly with the number of constituent elements, *n*. On the basis of the phase rule, the theoretical upper bound for the number of unique interfaces in multielement NPs grows quadratically with *n*. Interface arrangements resulting in these heterostructured NPs can lead to unprecedented properties (*1*), but we initially narrow our focus to the discovery of a special subset: biphasic, single-interface NPs (SINPs) in quaternary and higher-order chemical spaces. In two- or three-element NPs, expert intuition conditioned on knowledge of phase diagrams, crystal structures, and past observations can help target SINP compositions, but this intuition does not scale well to designing higher-order, chemically diverse SINPs, and researchers can greatly benefit from a feedback loop with machine learning. More broadly, we see SINPs as a critical first target for closed-loop, machine learning–driven design of heterostructured NPs because (i) the single interface enables facile electron microscopy characterization and, in turn, robust experimental results, and (ii) developing the capability for SINP discovery can further accelerate the development of NPs for applications that are influenced by interfaces, such as catalysis or plasmonics, by controlled deconvolution of interface-property relationships.

Sequential optimization strategies, such as BO, have started gaining attention in the chemical and physical sciences to solve high-dimensional and expensive optimization problems where factorial or intuition-driven experimental designs become infeasible (*5*, *25*–*30*). In BO, often used with a Gaussian process (GP) prior, the next experiment in a sequence of experiments is selected by an acquisition function that combines the predictions of the GP conditioned on past results with a decision-making strategy, balancing the exploration-exploitation trade-off, to maximize the gains toward an objective (*26*). We start the BO-driven SINP discovery effort by curating a dataset of 148 unique NP compositions from our reports, predating this study within the 8D Au-Ag-Cu-Co-Ni-Pd-Sn-Pt space, all synthesized and characterized under a consistent SBCPL-annealing STEM workflow that is adopted in this work (see Material and Methods and the Supplementary Materials) (*6*, *18*, *21*–*24*). Having this dataset is critical not only for improving the performance of BO in scheduling real experiments by conditioning the model on past data but also for designing and testing the BO strategy itself, before the actual costly deployment phase.

**Fig. 1.**
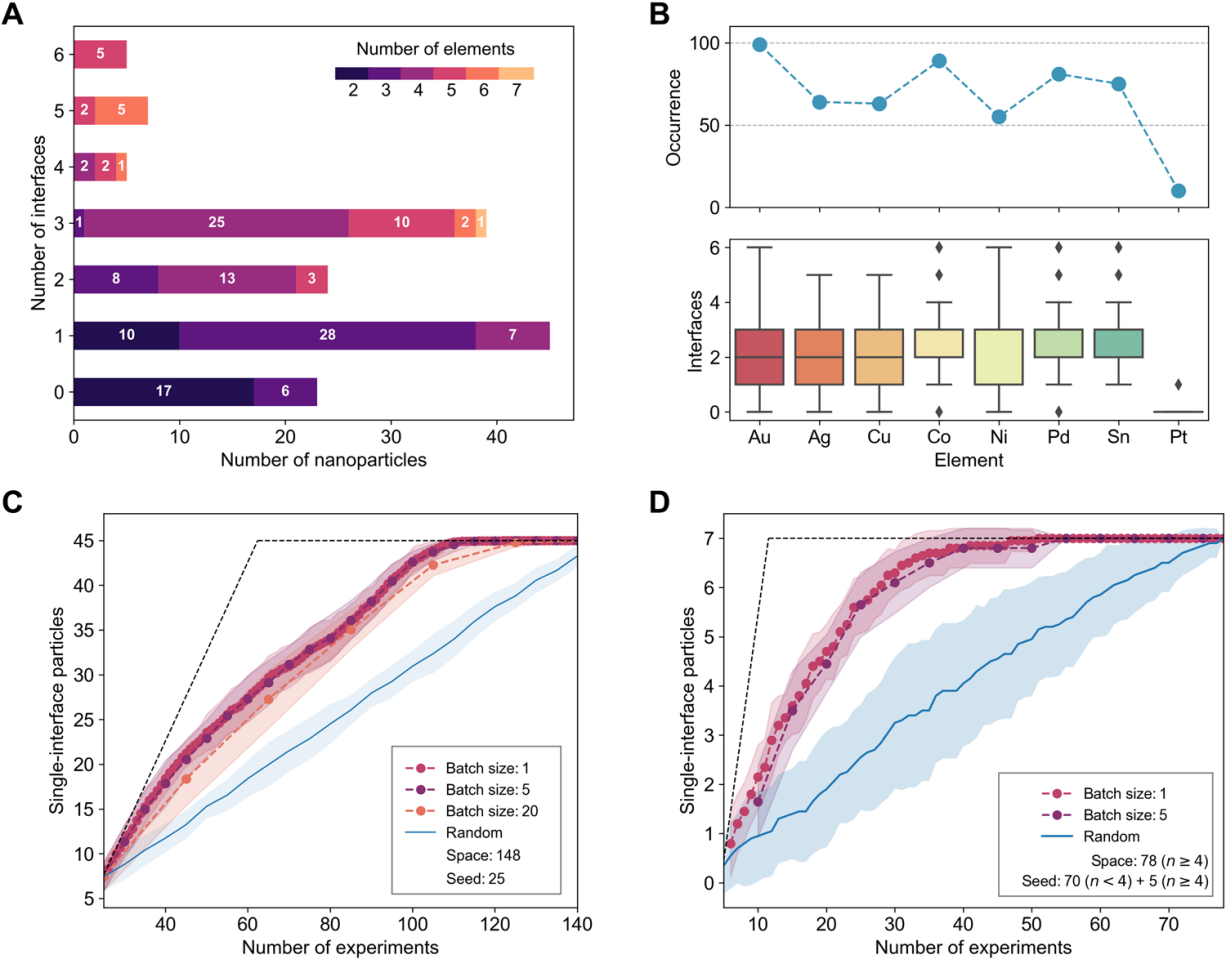
Statistics of the NP dataset and simulated optimization campaigns. (**A**) Distribution of the interface counts in the initial *N* = 148 NP dataset. Each interface count is broken down into segments on the basis of the number of elements making up the particle. (**B**) Occurrence of each of the eight elements in the initial dataset and the corresponding interface count box plots for each element. Performance results for the acquisition algorithm targeting single-interface particles, simulated using the existing dataset under two different scenarios in (**C**) and (**D**), at different batch sizes and compared to the random acquisition baseline. The performance (C) with a seed of 25 randomly selected particles from the space of 148 available, with the remaining space searched by the algorithm to find single-interface particles, and (D) with a seed made of 70 particles with *n* < 4 elements and 5 randomly selected from those with *n* ≥ 4, with the remaining space searched by the algorithm to find single-interface particles with *n* ≥ 4. Dashed lines show ideal performance limits and shaded regions show variability, limited to ± σ for visual clarity.

The chemical diversity and interfacial complexity of the NPs in this initial dataset are depicted in Fig. 1 (A and B), with the number of elemental components in each particle ranging from 2 to 7 and with the number of unique interface counts ranging from 0 to 6. In Fig. 1A, we observe that the dataset is dominated by two- to four-element NPs with zero to three interfaces, with higher interface counts appearing exclusively in higher-order systems, as expected. There are 45 SINPs in this dataset, limited to two- to four-element NPs, most abundant for ternary compositions. There are only seven quaternary SINPs and no pentanary or higher-order ones. Figure 1B shows that, except for Pt that exists in only a handful of NPs, there is no trivial relationship between the presence of a particular element in a particle and its interface count. While this dataset, to the best of our knowledge, is the largest compilation of microscopy-characterized alloy NPs, it still constitutes a highly sparse, researcher-driven sampling from independent studies, at compositions in highly disjointed subspaces of the Au-Ag-Cu-Co-Ni-Pd-Sn-Pt space. For example, approximately half of all composition pairs in the dataset share at most one common element, and of all possible two-, three-, four-, five-, six-, and seven-component spaces in the 8D design space, respectively, about 39, 64, 76, 80, 82, and 88% are empty, meaning they do not contain a single sample.

Hence, to overcome potential data inefficiencies in BO that would stem from sparsity and disjoint subspaces, we designed a domain-specific upper confidence bound (UCB) strategy and simulated its performance in BO for SINP discovery in the existing dataset before using it to help researchers in actual experiments. This UCB strategy transforms the NP compositions into a chemically informed and dynamically updated embedding space, axes of which are computable from composition and shared properties of elements (e.g., electronegativity) for all NPs rather than chemical identity of elements themselves (see Material and Methods for details). The first scenario that we simulated broadly targets the discovery of SINPs from the entire NP dataset using a seed of 25 randomly selected NPs (Fig. 1C). The second scenario targeted a more difficult search: discovery of seven SINPs among all 78 *n* ≥ 4 particles and a seed composed of all *n* < 4 particles and 5 randomly selected particles from the *n* ≥ 4 set (Fig. 1D). Under both scenarios, we confirmed that the new BO algorithm outperforms the “random” acquisition baseline by a substantial margin, without much depreciation in efficiency when forming small batches. Crucially, we found that the new domain-specific UCB strategy delivers up to around 10 and 40% acceleration relative to BO optimization with standard UCB methods over the compositional axis in the first and second SINP discovery scenarios, respectively (fig. S2). Overall, having built enough confidence in our BO methodology for SINPs, we next turned to actively deploying it to run a 4-week-long closed-loop experimental campaign.

### Closed-loop synthesis and characterization

**Fig. 2.**
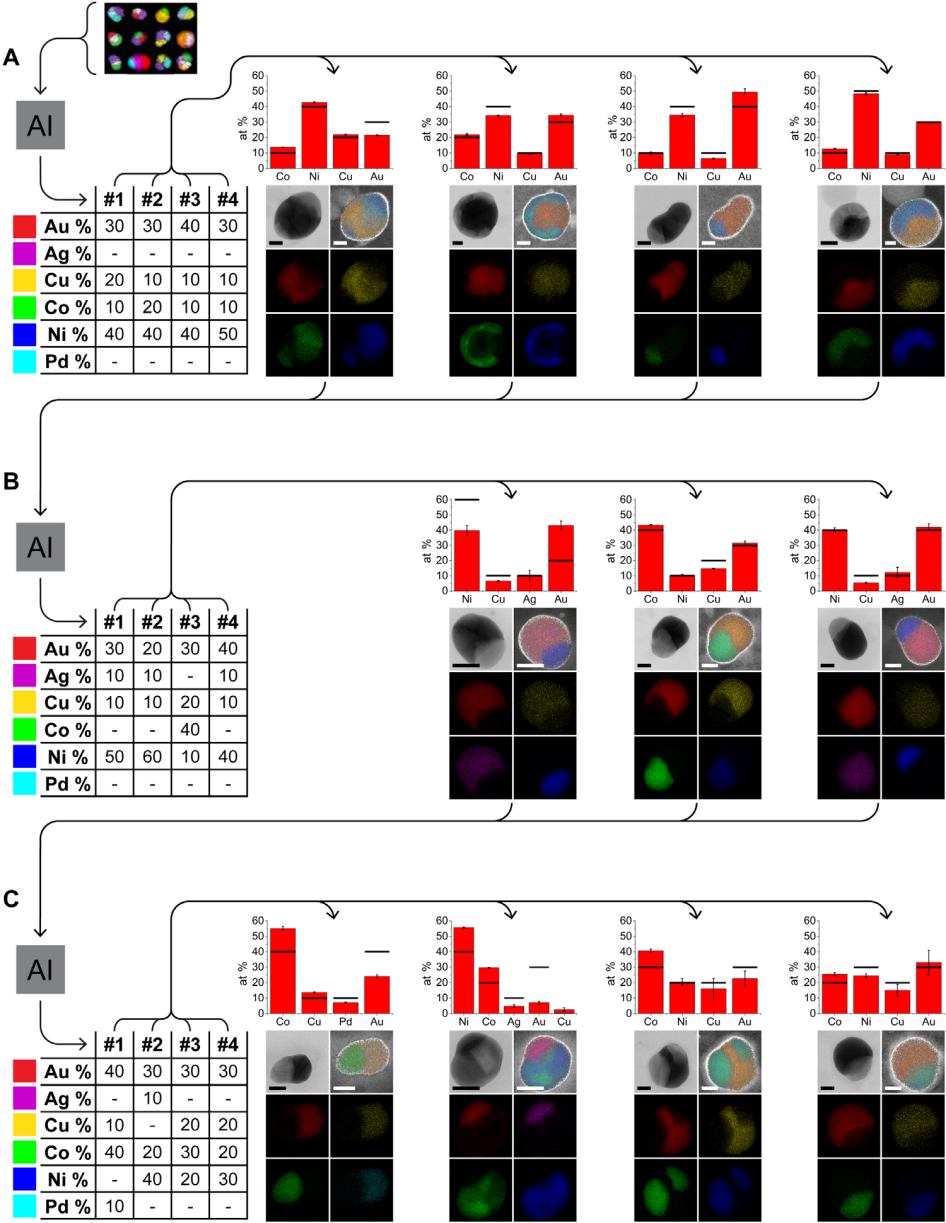
Closed-loop optimization for the discovery of quaternary metallic SINPs. (**A** to **C**) Iterations 1 to 3, respectively, of the closed feedback loop process. Starting from available input data, the top compositions suggested by the machine learning algorithm are tabulated and then synthesized by SPBCL. The resulting experimental compositions, as determined by STEM-EDS, are then fed back into the algorithm for the next set of predictions. Bar graphs show elemental compositions determined by EDS averaged across multiple NPs, with error bars showing the SD and black bars representing the suggested targets. Below are annular bright-field (ABF) images and EDS maps of a representative particle for each composition. Scale bars, 20 nm. Note that particle C2 contains a small amount of Cu contamination. at %, atomic %.

**Fig. 3.**
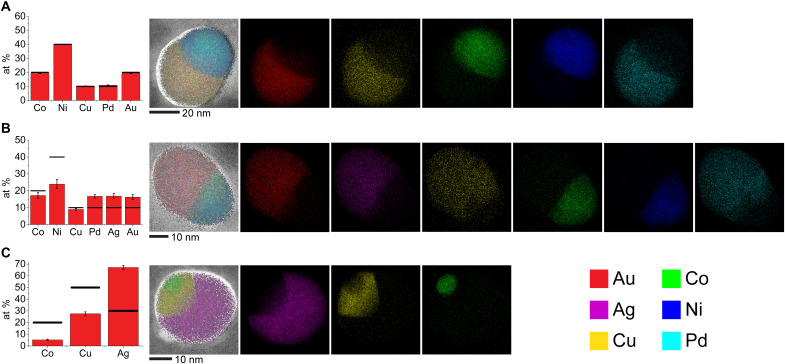
STEM-EDS analysis of exploratory NP compositions suggested by the optimization agent. Elemental compositions and EDS maps of a representative (**A**) quinary SINP, (**B**) senary SINP, and (**C**) ternary two-interface NP. Bar graphs show elemental compositions determined by EDS averaged across multiple NPs, with error bars showing the SD and black bars representing the suggested targets.

The workflow and results of the closed-loop iteration between experiment and BO algorithm for quaternary SINP discovery are described in Fig. 2, along with the results of a final exploratory step in Fig. 3, focusing on alternative composition-interface targets. Each round was initiated by a group of ranked target composition suggestions from the BO algorithm, which were then synthesized via a typical SPBCL NP synthesis experiment on a silicon nitride TEM membrane. The success of the synthesis experiments and the competency of the predictions were evaluated through STEM analysis. If the synthesis was successful, meaning single particles were formed and the resulting composition was near the suggested composition, three or more representative particles were quantitatively characterized by energy-dispersive x-ray spectroscopy (EDS) to determine their elemental compositions and distributions. The experimental data were then fed back into the algorithm database, and new composition suggestions were generated (see Material and Methods).

**Fig. 4.**
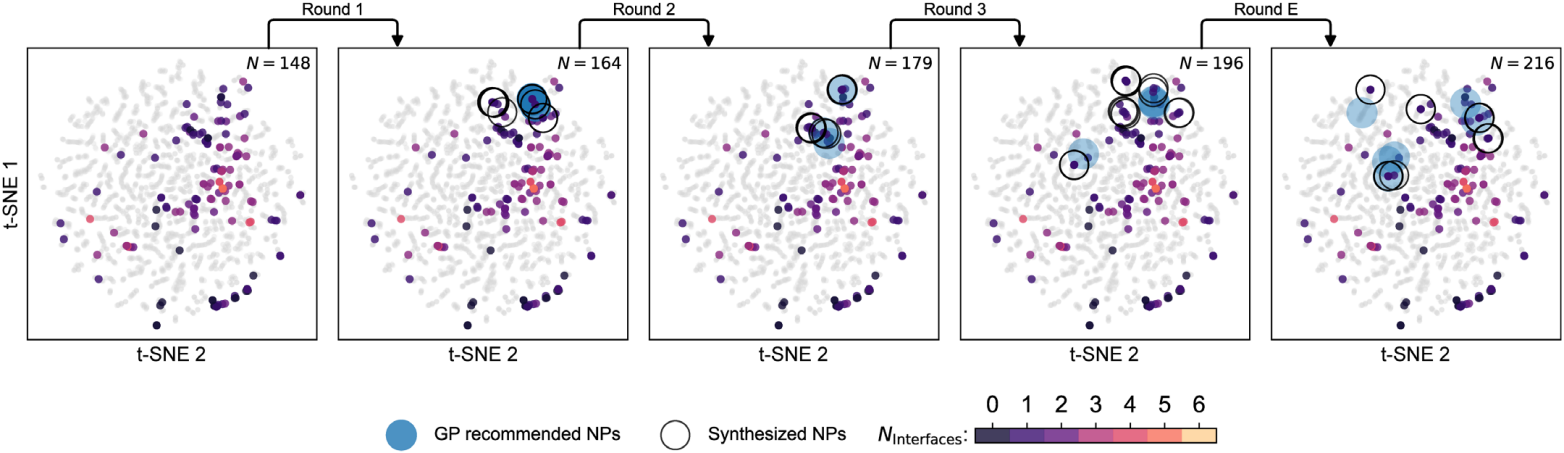
Characteristics of the 8D NP space and its BO-guided exploration for discovery of SINPs. This 2D projection of our composition-embedded BO design space (see Material and Methods) is generated using the *t-*distributed stochastic neighbor embedding (t-SNE) method. Gray circles represent the entire compositional search space of our 8D compositional grid (see Material and Methods). Experimental data (both initial and acquired in the loop) are color-coded with the measured interface counts. Acquisition suggestions by the algorithm (large open circles) and synthesized suggestions (large blue circles) are overlaid on the same projection to provide visual insights on how the search space is traversed with the aid of the BO algorithm. Round E corresponds to the “exploratory” search toward modified targets carried out upon completion of the closed-loop iteration for quaternary SINPs.

To first test the efficacy of the algorithm, we selected quaternary SINPs as our first target structure for reasons discussed above. Three rounds of suggestion and synthesis were performed in this space, wherein we synthesized the top 4 ranked suggested compositions, and of the 12 suggested compositions, 11 were successfully prepared and formed the target structure, with one synthetic failure. After only one round of suggestions, the algorithm moved away from simple permutations of an existing quaternary SINP composition and suggested novel compositions, which are visualized in Fig. 2. We find that the algorithm’s predictions are strongly influenced by the experimental feedback. For example, three of the four highest ranked suggestions in the second batch differed from the top 5 to 8 suggestions in the first batch, which would have become batch 2’s top 4 if the rankings were not influenced by the new results. Figure 4 provides an alternative view of the 8D composition space by projecting the high-dimensional compositional embedding space used in our BO method to a more intuitive 2D visualization (see Material and Methods). First, we observe that the data in our initial dataset are distributed reasonably well throughout the large, high-dimensional design space we are searching. We also find that the algorithm targets a certain part of the chemical space in the first two rounds, exploiting regions near existing SINPs, although in the third and exploration rounds (explained below in more detail), it ventures out of that region. On the basis of the projections of our EDS measured average compositions on the same 2D representation in Fig. 4, apart from a few visible deviations, we can track the BO suggestions in the chemical design space reasonably well.

Typical SINPs have an ellipsoidal shape with a single, straight interface whose location depends on the relative amounts of material present in the constituent phases (see, for example, Fig. 2B); however, particularly in larger particles, this desired structure is not always achieved. During particle coarsening, it is possible for smaller particles to attach to each other at different locations, leading to particles whose second phase is discontinuous or inhomogeneously distributed (Fig. 2, A1). Note that the discontinuous phase is always the one consisting of the lower-mobility metals, such as Ni and Co, which naturally take longer to form thermodynamic structures during low-temperature annealing. As a result, we hypothesized that these structures could gradually be transformed into their thermodynamic analogs through prolonged annealing. During annealing, the structure gradually approaches those that are thermodynamically favored (fig. S3). Therefore, we conclude that these particles can, for the purposes of identifying their interfaces, be treated as equivalent to ones already exhibiting the desired thermodynamic structures.

After the first three iterations of the model system resulted in competent suggestions, we explored several unprecedented phase spaces to test the capabilities of the algorithm (Fig. 3). In particular, we changed our targets to ones that had no analogs in the existing dataset (i.e., quinary and senary SINPs), forcing the algorithm to extrapolate from the existing data. Starting with quinary SINPs, the algorithm’s top 5 suggested candidates were all variations of AuCuCoNiPd; however, due to a scientific curiosity and a desire to maximize the search space, we selected the two highest ranked variations of AuCuCoNiPd, the two highest ranked variations of AuAgCuCoNi, and one variation of AuCuCoNiPd that was markedly different than the other suggested compositions. Again, in each case, the algorithm provided competent suggestions (Fig. 3A and fig. S4), although there was no existing data to draw from. A related previously reported AuAgCuCoNi composition was a triphasic, two-interface particle (*22*), yet the suggested compositions reported here all yielded the target biphasic structure, highlighting the capabilities of the algorithm to explore different regions of phase space.

Next, we went multiple steps further by targeting both a senary SINP and two-interface particles. We selected the highest ranked suggestion for the senary SINP from the BO model conditioned on all data that we collected up to this point, and we successfully synthesized, to the best of our knowledge, the most chemically complex single-interface metallic NP ever prepared (Fig. 3B). In our attempt to synthesize two-interface particles, we selected the highest ranked permutation of an AgCuCo composition (again with BO conditioned on all past data), which successfully formed the target structure (Fig. 3C).

**Fig. 5.**
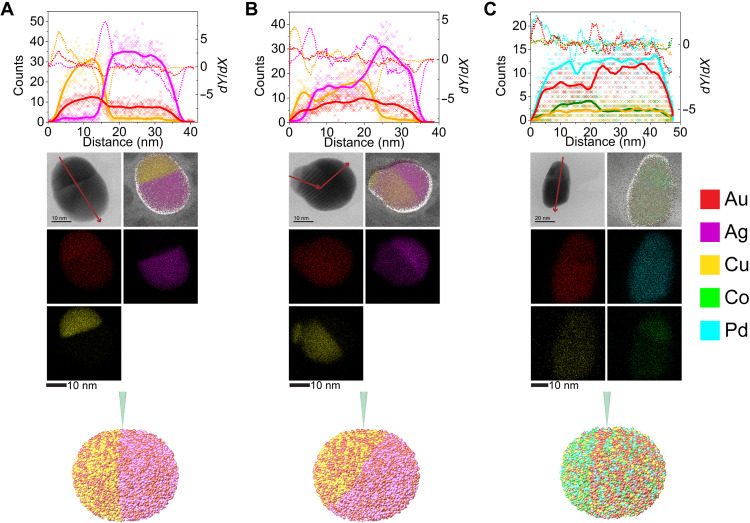
STEM-EDS characterization of interfaces in different biphasic NPs. EDS line profiles taken along the red arrows in the ABF images, corresponding EDS maps, and schematic models of particle orientation with respect to the electron beam of (**A** and **B**) Au_10_Ag_30_Cu_60_ and (**C**) Au_30_Cu_10_Co_20_Pd_40_ NPs. Crosses and solid curves represent raw and smoothed data, respectively, and dotted curves are the corresponding gradients.

During this final effort to explore two-interface NPs, a particular high-ranking suggestion in the Au-Ag-Cu space caught our attention as it conflicted with our intuition and bulk phase diagrams and, hence, would serve as a case study for understanding the underlying algorithmic and experimental causes of these conflicts. As shown in Fig. 5A, we confirmed that NPs synthesized at this nominal composition were off the two-interface target. Further inspection revealed that the GP algorithm relied on two nearby points in this ternary space in our dataset with a larger than average uncertainty in composition and phase structure. Characterization of numerous AuAgCu particles reveals that the inherently random orientation of the synthesized NPs on a surface can easily lead to ambiguities in characterization (Fig. 5, A and B) when interfaces are not oriented along the optical axis of the microscope, making a region of two overlapping phases indistinguishable from a third phase by imaging or EDS. Moiré fringes can indicate phase overlap (e.g., Fig. 5B and fig. S3C), but their absence does not prove the existence of another phase. Peaks in gradients of compositional line profiles can help locate interfaces [e.g., one versus two peaks in Fig. 5 (A and B), respectively], but by aligning its interface with the optical axis, the particle in Fig. 5B is identified as having a single interface (fig. S3E). Overall, without reliable statistical averages over the particle orientations or additional characterization techniques, particles such as those in Fig. 5 (A and B) can easily be interpreted as having more than one type of interface, leading to ambiguity in input data. We confirmed that, upon removal of the two such suspect AuAgCu points from our NP dataset, the BO algorithm no longer suggests any AuAgCu compositions for a two-interface target (not one in the top 100 suggestions in ternary space). The addition of the new AuAgCu data does not immediately eliminate this off-target suggestion, indicating that it would require more BO iterations, and hence, keeping the researcher in the loop for the identification and elimination of “bad” data is a more cost-effective remedy.

We emphasize, however, that homogeneously distributed elements in high-order NPs can bring about more and nearly intangible interfaces (Fig. 5C). In certain cases, large percentages of miscible elements lead to these “soft” interfaces, wherein the transition from one phase to the other is less obvious. The examples of AuAgCu (Fig. 5A) and AuCuCoPd (Fig. 5C) illustrate the “hard” and soft interface extremes, respectively. This observation highlights shortcomings in defining what constitutes an interface. Clearly, a discontinuous change in crystal structure is an interface, but for coherent interfaces with continuous changes in structure, there is no universally accepted answer for exactly how steep a gradient in composition and/or lattice parameter must be to be classified as such. For the purposes of this work, all interfaces are classified equivalently, but the nature of the interface poses an interesting structural motif that, with the requisite characterization capabilities, could be investigated in more detail.

Considering the success of our closed-loop implementation and our in-depth inspection of the AuAgCu case, we identify several opportunities for improvement for BO-driven NP-interface discovery: (i) acquiring reliable data for building predictive models for BO, (ii) keeping the “researcher in the loop” when the experiment throughput allows for inspection of suggestions and interventions, and (iii) improving the algorithmic capabilities in all stages of BO. Methods such as 4D STEM would enable complete mapping of crystal structure throughout individual particles and, therefore, remove ambiguities otherwise present in EDS maps and allow for simultaneous identification of composition, morphology, and crystal structure. While researchers can always intervene, algorithmic improvements that would bring ambiguous input data to their attention would make interventions and, in turn, overall BO more efficient. Although our domain-specific, composition-embedded BO strategy improves upon more standard UCB methods, the optimization methodology certainly has room for improvement, particularly in active research areas (*31*, *32*) such as accounting for the uncertainty in input compositions and targets in BO.

## DISCUSSION

In this work, we have demonstrated that machine learning–assisted optimization is a viable approach to identifying nanostructures with target morphological qualities, even with limited datasets. This platform allowed us to synthesize new and complex materials, including, to the best of our knowledge, the most complex biphasic NP ever made, with success rates far greater than random selection (18 of 19) and, in more complex circumstances, greater than a keen chemical intuition. The limitations for expansion of such a design feedback loop to higher-throughput synthesis and experimentation, and into more complex properties, such as catalytic activity or stability, are almost exclusively experimental. In this work, we synthesize one material at a time using dip-pen nanolithography (DPN) to accommodate the electron microscopy characterization; parallelization of SPBCL to synthesize thousands or millions of unique materials in a single experiment is possible and has been demonstrated (*2*). As we develop new characterization techniques to extract more information from megalibraries of nanomaterials at higher throughputs, and as targeted properties become increasingly more complex in future work, the benefits of using such a machine learning aid to guide the search will increase exponentially, as the capabilities of an algorithm can scale unlike those of human intuition.

## MATERIALS AND METHODS

### Materials and chemicals

Chloroauric acid hydrate (HAuCl_4_ *x*H_2_O; ≥99.9% trace metals basis), cobalt(II) nitrate hexahydrate [Co(NO_3_)_2_ 6H_2_O; 99.999%], copper(II) nitrate hydrate [Cu(NO_3_)_2_ *x*H_2_O; 99.999%], nickel(II) nitrate hexahydrate [Ni(NO_3_)_2_ 6H_2_O; 99.999%], palladium(II) nitrate dihydrate [Pd(NO_3_)_2_ 2H_2_O; ~40% Pd basis], silver nitrate (AgNO_3_; anhydrous, ≥99.999% trace metals basis), nitric acid (HNO_3_; 70%, redistilled, ≥99.999% trace metals basis), hexamethyldisilazane (HMDS; 99.9%), and hexane (anhydrous, 95%) were purchased from Sigma-Aldrich. Poly(ethylene oxide)-*block*-poly(2-vinylpyridine) (PEO-*b*-P2VP; *M*_n_ = 2.8-*b*-1.5 kg/mol, polydispersity index = 1.11) was purchased from Polymer Source Inc. DPN 1D pen arrays (type M, without a gold coating) were purchased from Advanced Creative Solutions Technology LLC. Silicon nitride membranes for TEM (amorphous, thickness of 15 nm) were purchased from Ted Pella Inc.

### NP synthesis

Silicon nitride membranes were functionalized with HMDS by vapor deposition in a closed chamber containing an HMDS/hexanes mixture (1:1 v/v) overnight at room temperature; rinsed with toluene, isopropanol, and acetone; and dried with N_2_. Precursor inks were prepared by dissolving PEO-*b*-P2VP and metal precursors at desired ratios in deionized water, such that the final polymer concentration was 5.0 mg/ml and the total metal to pyridyl group molar ratio was 1:32. The inks were then acidified with 2.0 μl of 1.0 M HNO_3_ per milliliter of ink and left to shake overnight in the dark. DPN pen arrays were plasma-treated at 30 W for 60 s and mounted onto an atomic force microscopy (Park Systems XE-150) in a chamber kept at 20°C and a relative humidity of 80%. Pen arrays were inked by bringing the pen array into contact with a dropcast ink reservoir, and nanoreactors were deposited onto the functionalized silicon nitride membranes by repeatedly bringing the cantilever tips in contact with the surface. Samples were thermally annealed in a tube furnace with an H_2_ flow of 100 standard cubic centimeters per minute using the following protocol: ramped to 150°C at 2.2°C/min, held at 150°C for 6 hours, ramped to 300°C at 2.5°C/min, held at 300°C for 6 hours, ramped to 500°C at 3.3°C/min, held at 500°C for 12 hours, and then cooled to room temperature.

### NP characterization

All STEM and EDS characterization was carried out on a JEOL JEM-ARM200CF equipped with a cold field-emission gun operated at 200 kV, a CEOS hexapole probe aberration corrector, and two EDS detectors using a JEOL beryllium holder. High-angle annular dark-field (HAADF) and annular bright-field (ABF) images were acquired with a convergence angle of 20.6 mrad and a collection angle of 68 to 280 mrad (HAADF) or 7.5 to 17 mrad (ABF). The Lα peaks of Ag, Au, and Pd and the Kα peaks of Co, Cu, and Ni were used for EDS quantification and mapping. Elemental maps were generated from at least 100 frames with a pixel dwell time of 20 μs and processed to show net x-ray counts using a 3 × 3 kernel with one full pixel overlap. EDS quantification was carried out using the standardless Cliff-Lorimer correction method with the open source Python library HyperSpy (*33*). For each composition, multiple NPs were quantified to obtain a representative average composition.

### BO algorithm design

Our BO strategy derives from the GP-UCB method (*34*). In GP-UCB, the next point of acquisition ***x****_t_* at step *t* from a decision space *D* (***x*** ∈ *D*) in a sequence of experiments is decided as $x_{t}=\text{argmax}[\mu_{t-1}(x)+\sqrt{\beta_{t}}\sigma_{t-1}(x)]$, which provides an exploration-exploitation trade-off by combining the predictions, μ(***x***), with the predicted uncertainties σ(***x***) weighted by $\sqrt{\beta_{t}}$. Decisions made using this approach after the first selection in a batch are made under information poor conditions and may lead to over- or underexploration of parts of *D*, which can, in part, be mitigated by reconditioning the GP using the past selections in the batch (*35*, *36*) to update the uncertainty estimates.

In our context, NP optimization is to be carried out in an 8D space where ***x*** ≔ [*c*_Ag_, *c*_Au_, …, *c*_Sn_] and *c_i_* is the molar fraction of element *i*. In practice, many compositions ***x*** are confined to a lower-dimensional subspace of *D*, and hence, information from fully or partially disjoint subspaces (e.g., Au-Cu-Pd versus Ag-Ni-Sn) cannot be used efficiently in BO. We therefore map ***x*** to an embedding space ***r*** using a transformation *g* that converts the design axes from elemental compositions to descriptors derived from stoichiometry and properties of elements, hence shared across all NPs. Because the relevance of descriptors is not known a priori, for each batch *B*, *g* is parameterized by data available up to that point (*t*′ being the latest measurement; *t*′ < *t*) and used to dynamically generate the embedding space ***r****_B_*(***x***) = *g*(***x***; *x*_1_, *x*_2_, …, *x*_*t*^′^_) from ***x***. Thus, to compose batch *B*, acquisitions are decided as

$$r_{B,t}=\text{argmax}[\mu_{t-1}(r_{B})+\sqrt{\beta_{t}}\sigma_{t-1}(r_{B})]$$

and repeated by reconditioning GP with past selections until the desired batch size is reached. To recast our target-interface-count problem as maximization, we define $y_{t}≔-|y_{t}^{\prime }-n|$ and *n* is the targeted interface count (e.g., *n* = 1 for SINPs).

We parameterize *g* for a batch *B* as follows: (i) create a large set of *m* descriptors for each composition *x*_1_, *x*_2_, …, *x*_*t*^′^_ measured until *B*; a *t*′ × *m* matrix of scalars; (ii) standardize each column; and (iii) find a transformation to a more compact *N*_PCA_-dimensional embedding space using principal components analysis (PCA). This parameterized *g* is then used to transform the search space ***x*** to ***r****_B_* for batch *B*. *N*_PCA_ is tuned a priori with the aim of finding a small enough space (*N*_PCA_ ≪ *m*), where GP provides sufficient accuracy and BO can be performed efficiently.

### Hyperparameters of the optimization framework

We use the compositional descriptors (*m* = 132) described by Ward *et al.* (*37*, *38*), which are derived from statistical aggregations of stoichiometries and various elemental properties, in our implementation of *g*. We select *N*_PCA_ = 20 for the active closed-loop experiment-algorithm iterations on the basis of fig. S1 (see Supplementary Text). We use the radial basis function kernel $k(r,r\prime )=\sigma^{2}\text{exp}(-\frac{1}{2\ell^{2}}{\left\|r-r\prime \right\|}^{2})$ with all GP models, where σ, ℓ, and the noise variance are optimized using the *bfgs* algorithm. Parameter β*_t_* is dynamically set using the theorem 1 of (*34*) with a scaling factor of 0.05. We use the GPy library for implementation of GPs in BO, with the rest of the machine learning and data transformation steps handled through the scikit-learn library. The BO workflow is carried out using the camd python library.

### Compositional search space design

For the closed-loop optimization, we generate a discretized search space *D* by creating a grid of compositions with 10% increments on each composition axis, i.e., Ag, Au, Cu, Co, Ni, Pd, and Sn. We use data from Pt-bearing particles in the BO seed, and hence, Pt is also included as a composition axis in *D*, but these particles are not targeted in the search itself. We remove compositions from the search space that are closer than 5% in at least one of the composition axes to those that already exist in our initial NP dataset and those that are sent to and received from experimental workflow in each iteration.
