# Supplementary material for: Machine learning–accelerated design and synthesis of polyelemental heterostructures
Source: Sci Adv. 2021 Dec 22;7(52):eabj5505. doi: 10.1126/sciadv.abj5505 (PMC8694626; doi:10.1126/sciadv.abj5505)
Supplement: Supplementary file 1 — Supplementary Text Figs. S1 to S16 [file sciadv.abj5505_sm.pdf]

Science Advances
AAAS

Supplementary Materials for

Machine learning–accelerated design and synthesis of polyelemental heterostructures

Carolin B. Wahl, Muratahan Aykol, Jordan H. Swisher, Joseph H. Montoya, Santosh K. Suram, Chad A. Mirkin*

*Corresponding author. Email: chadnano@northwestern.edu

Published 22 December 2021, *Sci. Adv.* **7**, eabj5505 (2021)
DOI: 10.1126/sciadv.abj5505

This PDF file includes:

Supplementary Text
Figs. S1 to S16

Supplementary Text

We show in fig. S1 that the dynamic embedding strategy we use in this work improves predictive accuracy for our target for both a linear type (ElasticNet) and a GP(RBF) model compared to modeling with composition axes only. Based on the validation study in fig. S1, we choose $N_{PCA} = 20$ for the active closed-loop experiment-algorithm iterations. More importantly, we show in fig. S2 that the UCB strategy described above provides higher BO performance in our simulations for SINP discovery using past data over standard GP-UCB strategies at the sequential limit as well as in small batches (*34, 36*), ranging from a marginal improvement of about 10% in a data-rich scenario to almost 40% in a more difficult (less frequent) target scenario.

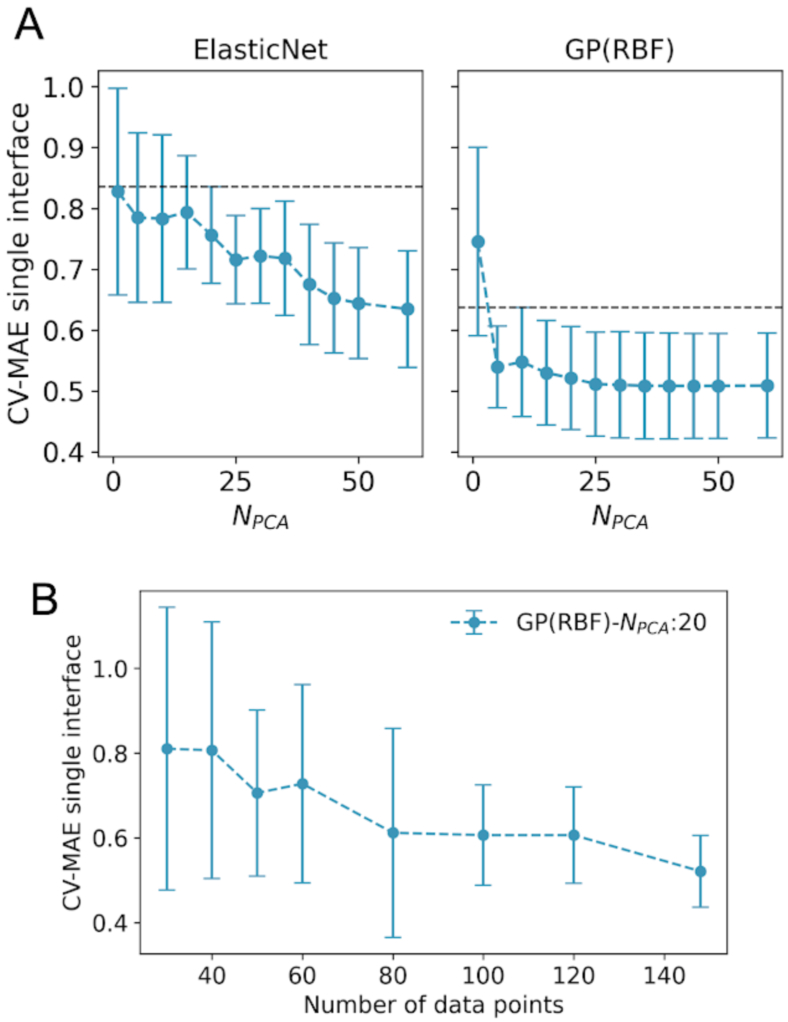

Fig. S1.

Testing and validation of the machine learning strategies in predicting the optimization target. (A) Cross-validated (CV) mean absolute error (MAE) scores as a function of the number of principal components in feature vectors for two regression algorithms based on a regularized linear model (ElasticNet) and Gaussian Process (GP) with a radial basis function (RBF) kernel. Error bars show one standard deviation in scores among each fold in a 10-fold CV procedure and dashed-lines show the MAE scores of the same algorithms trained using elemental compositions only, instead of the principal components of the statistically featurized compositions. (B) Learning performance as a function of the number of datapoints for the GP(RBF) with $\boldsymbol{N_{PCA}} = \mathbf{20}$ principal components in feature vectors, error bars identical to those in panel (A).

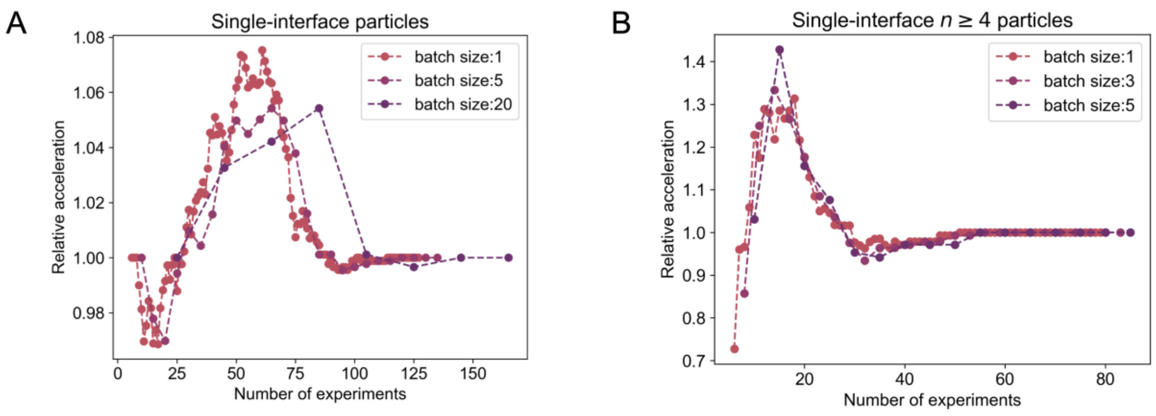

Fig. S2.

Relative acceleration in BO with domain-specific UCB designed in this work relative to standard UCB. (A) and (B) correspond to first and second SINP discovery scenarios described in the manuscript. Relative acceleration is defined as the ratio of average number of discoveries (simulated over 20 initializations) in each type of campaign at a given number of experiments. We see close to a 10% and 40% increase in peak efficiency through the course of scenarios pertaining to (A) and (B), respectively, with the UCB we designed, relative to the standard UCB.

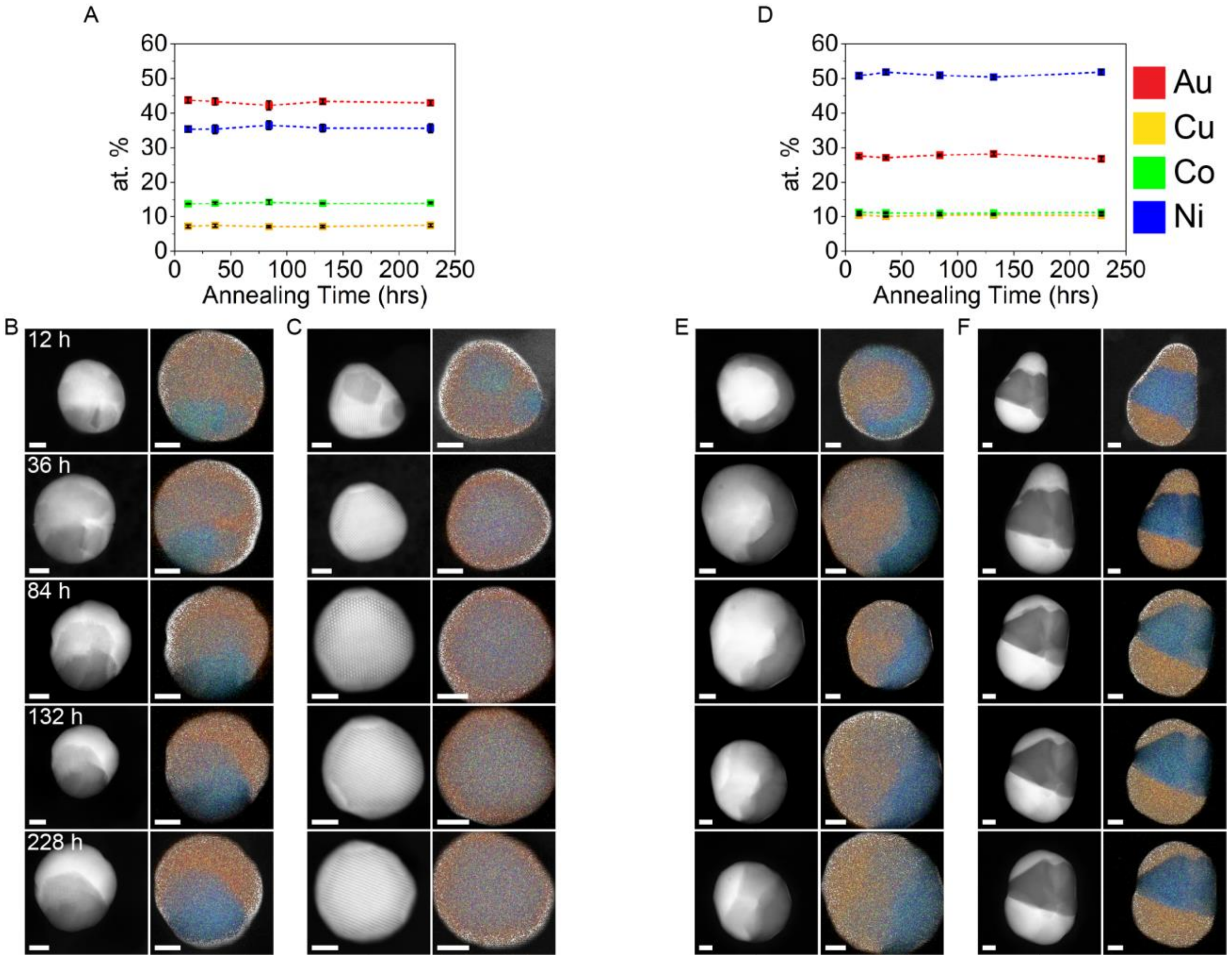

Fig. S3.

Evolution of quaternary SINPs during prolonged annealing. Plot of the elemental composition of (A) $Au_{40}Cu_{10}Co_{10}Ni_{40}$ and (D) $Au_{30}Cu_{10}Co_{10}Ni_{50}$ NPs as a function of annealing time. Error bars represent standard deviations. HAADF images and EDS maps of (B,C) $Au_{40}Cu_{10}Co_{10}Ni_{40}$ and (E,F) $Au_{30}Cu_{10}Co_{10}Ni_{50}$ NPs annealed at 500 °C for up to 228 h and imaged at different timepoints. Scale bars: 10 nm.

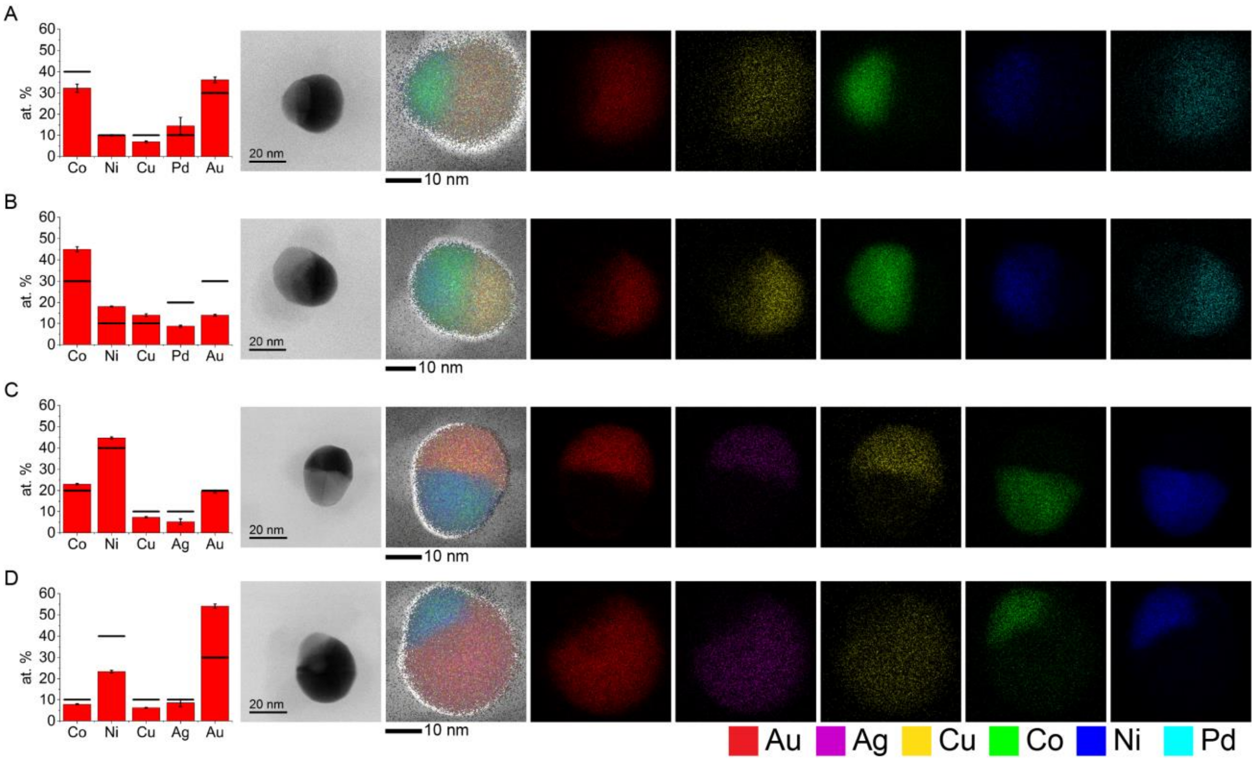

Fig. S4.

STEM-EDS analysis of quinary SINP compositions. (A-D) Elemental compositions, ABF images, and EDS maps of representative quinary SINPs suggested by the optimization agent. Bar graphs show elemental compositions determined by EDS with error bars showing the standard deviation, where the black bars represent the suggested targets.

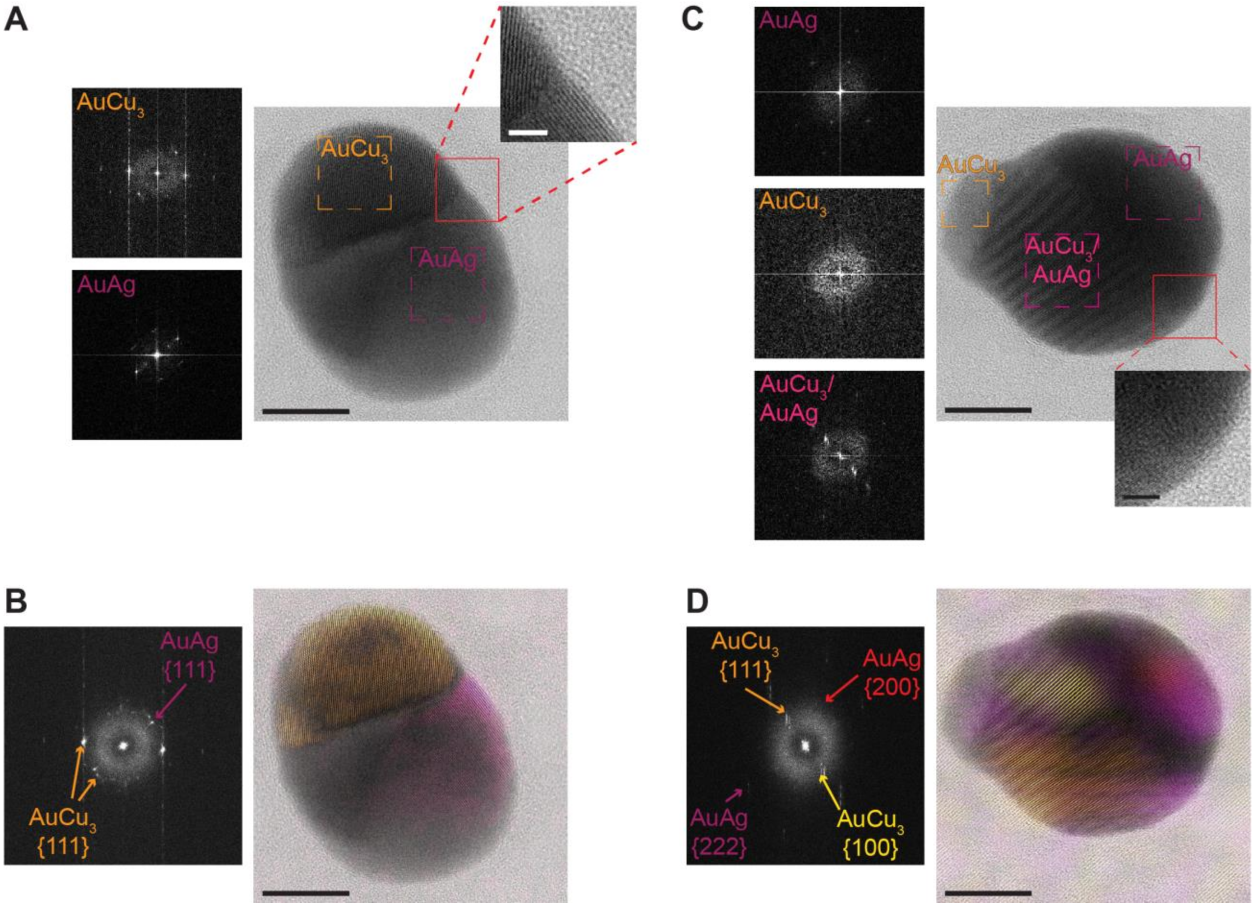

Fig. S5.

Phase structure of the AuAgCu NPs shown in Fig. 5A and 5B. (A) ABF image of the AuAgCu NP in Fig. 5A (scale bar: 10 nm) with an enlarged image of the interfacial region (scale bar: 2 nm). Fast Fourier transforms (FFTs) of the dashed box regions show crystal structures corresponding to $AuCu_3$ and a AuAg alloy. EDS spectra from the same regions show compositions of 28% Au, 72% Cu and 19% Au, 81% Ag, respectively. (B) Overlay of two sets of $AuCu_3$ {111} and one set of AuAg {111} planes extracted from the FFT of the image in (A). (C) ABF image of the AuAgCu NP in Fig. 5B (scale bar: 10 nm) with an enlarged image of the interfacial region between the pure AuAg phase and the overlap region (scale bar: 2 nm). FFTs of the highlighted regions show crystal structures corresponding to $AuCu_3$ and a AuAg alloy, as well as their combination when both structures coexist. EDS spectra from the same regions show compositions of 30% Au, 70% Cu in the pure $AuCu_3$ region, 20% Au, 80% Ag, in the pure AuAg region, and 22% Au, 39% Ag, 39% Cu in the overlap region. (D) Overlay of $AuCu_3$ {111} and {100} and AuAg {222} and {200} planes visible in different regions of the multicrystalline particle, extracted from the FFT of the image in (D), highlighting the coexistence of both $AuCu_3$ and AuAg phases in the central region of the particle. The AuAg {222}and {200} planes were chosen over the (also visible) {111} and {100} planes as they are more easily distinguished from the $AuCu_3$ planes.

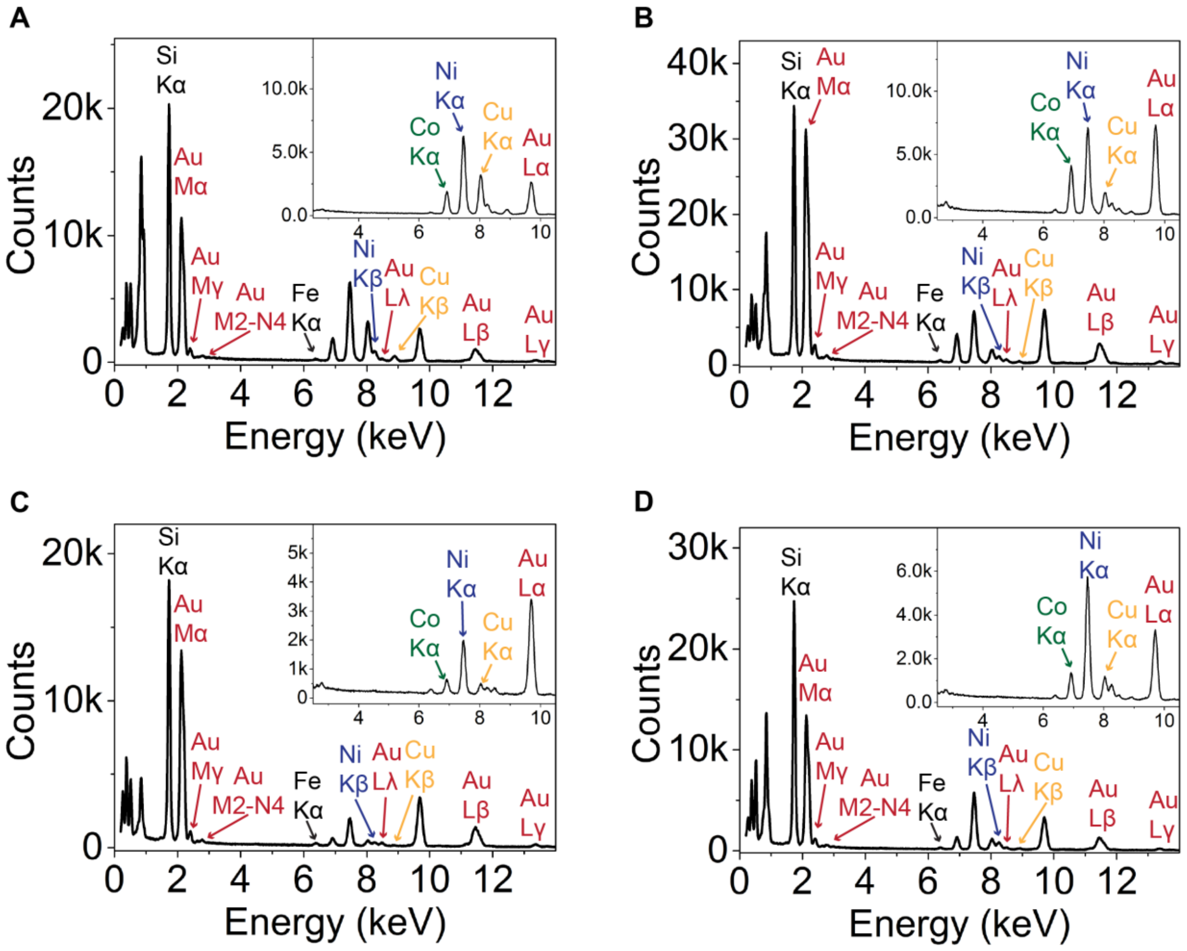

Fig. S6.

EDS spectra of the quaternary AuCuCoNi SINPs in Fig. 2A 1-4. (A) $Au_{30}Cu_{20}Co_{10}Ni_{40}$, (B) $Au_{30}Cu_{10}Co_{20}Ni_{40}$, (C) $Au_{40}Cu_{10}Co_{10}Ni_{40}$, and (D) $Au_{30}Cu_{10}Co_{10}Ni_{50}$. Insets are enlarged regions of interest showing the peaks used for quantification. Unlabeled peaks in the 0-1 keV region: C Kα (277 eV), N Kα (392 eV), O Kα (525 eV), Co Lα (776 eV), Ni Lα (851 eV), and Cu Lα (930 eV). The L-lines of first-row transition metals are too close in energy to be resolved and appear as a single peak.

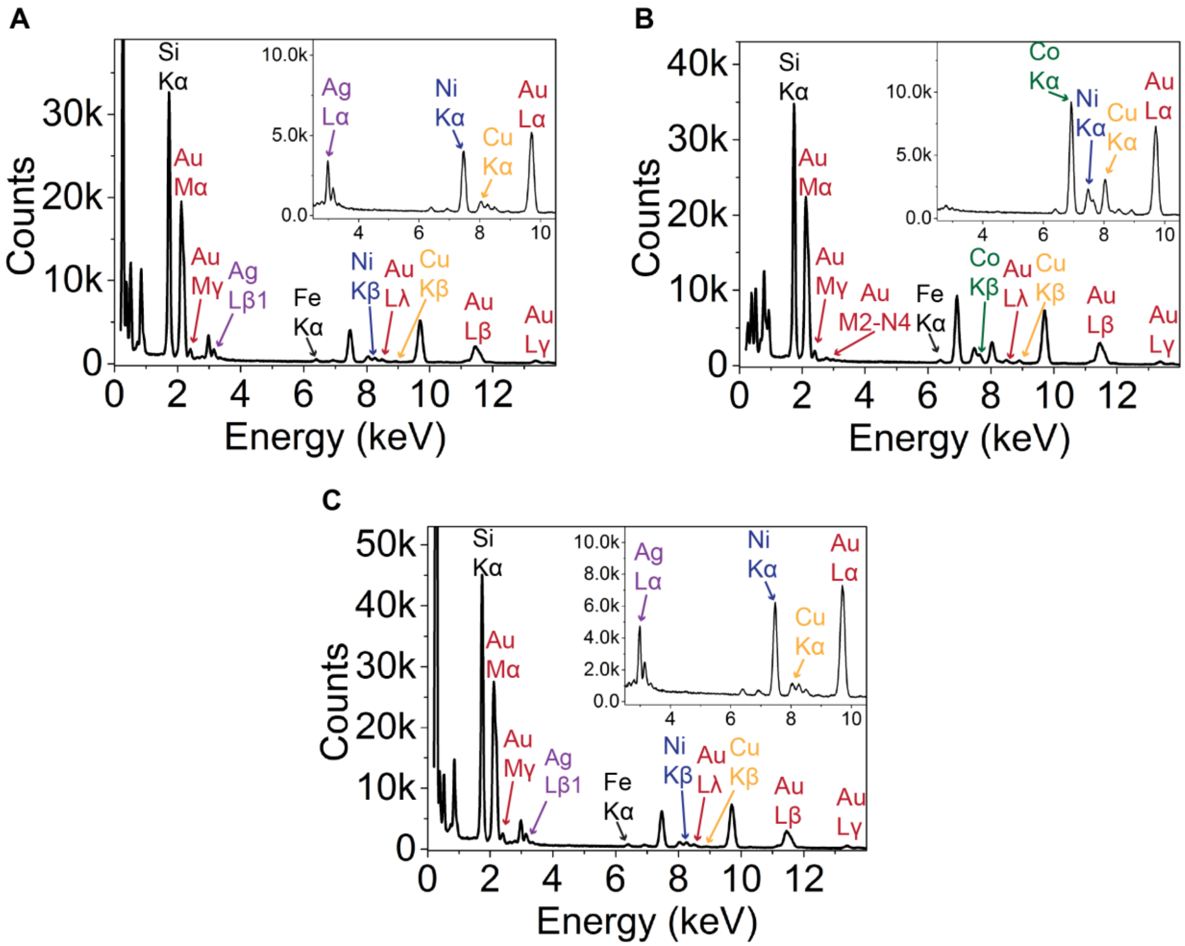

Fig. S7.

EDS spectra of the quaternary SINPs in Fig. 2B 2-4. (A) $Au_{20}Ag_{10}Cu_{10}Ni_{50}$, (B) $Au_{30}Cu_{20}Co_{40}Ni_{10}$, and (C) $Au_{40}Ag_{10}Cu_{10}Ni_{40}$. Insets are enlarged regions of interest showing the peaks used for quantification. Unlabeled peaks in the 0-1 keV region: C Kα (277 eV), N Kα (392 eV), O Kα (525 eV), Co Lα (776 eV, in B), Ni Lα (851 eV), and Cu Lα (930 eV).

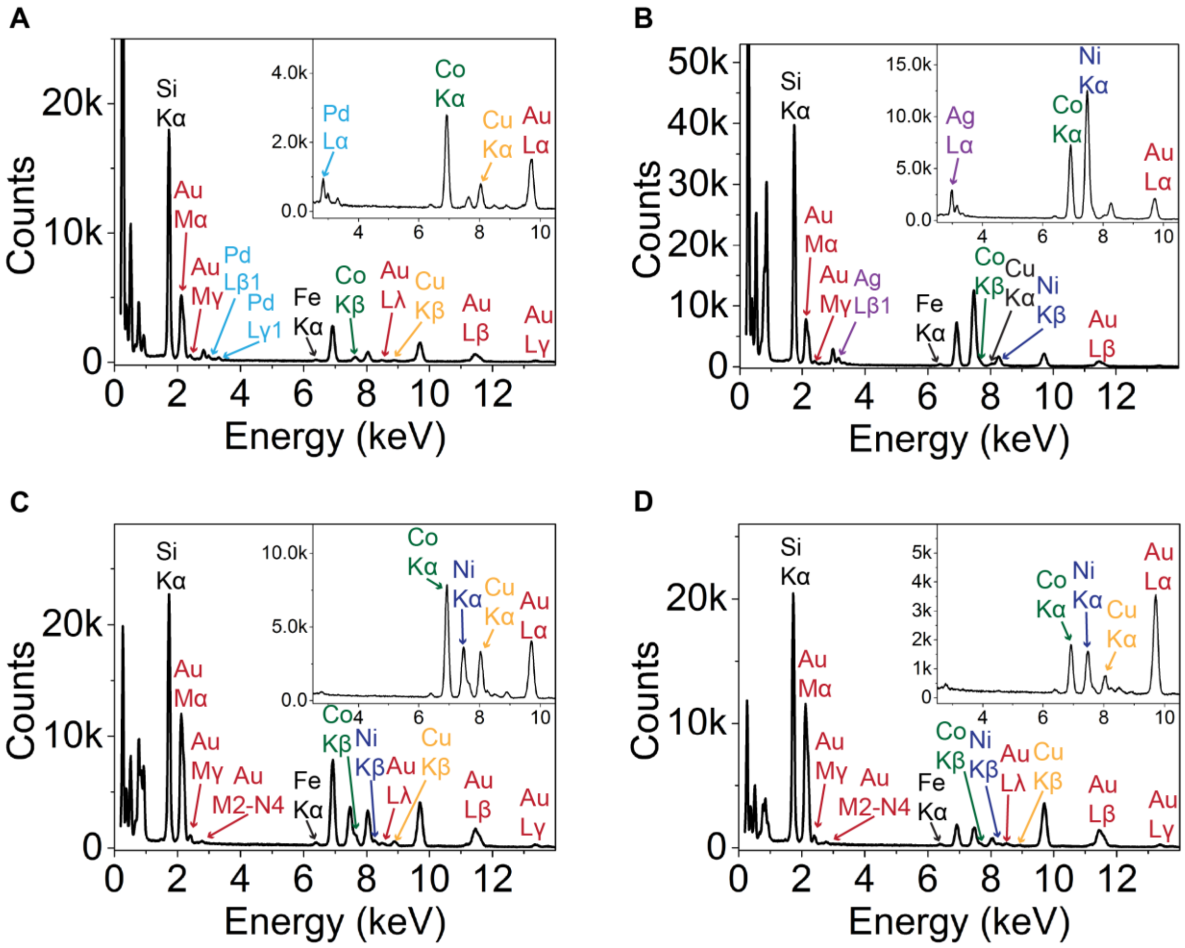

Fig. S8.

EDS spectra of the quaternary SINPs in Fig. 2C 1-4. (A) $Au_{40}Cu_{10}Co_{40}Pd_{10}$, (B) $Au_{30}Ag_{10}Co_{20}Ni_{40}$, (C) $Au_{30}Cu_{20}Co_{30}Ni_{20}$, and (D) $Au_{30}Cu_{20}Co_{20}Ni_{30}$. Insets are enlarged regions of interest showing the peaks used for quantification. Unlabeled peaks in the 0-1 keV region: C Kα (277 eV), N Kα (392 eV), O Kα (525 eV), Co Lα (776 eV), Ni Lα (851 eV, in B-D), and Cu Lα (930 eV, in A,C,D).

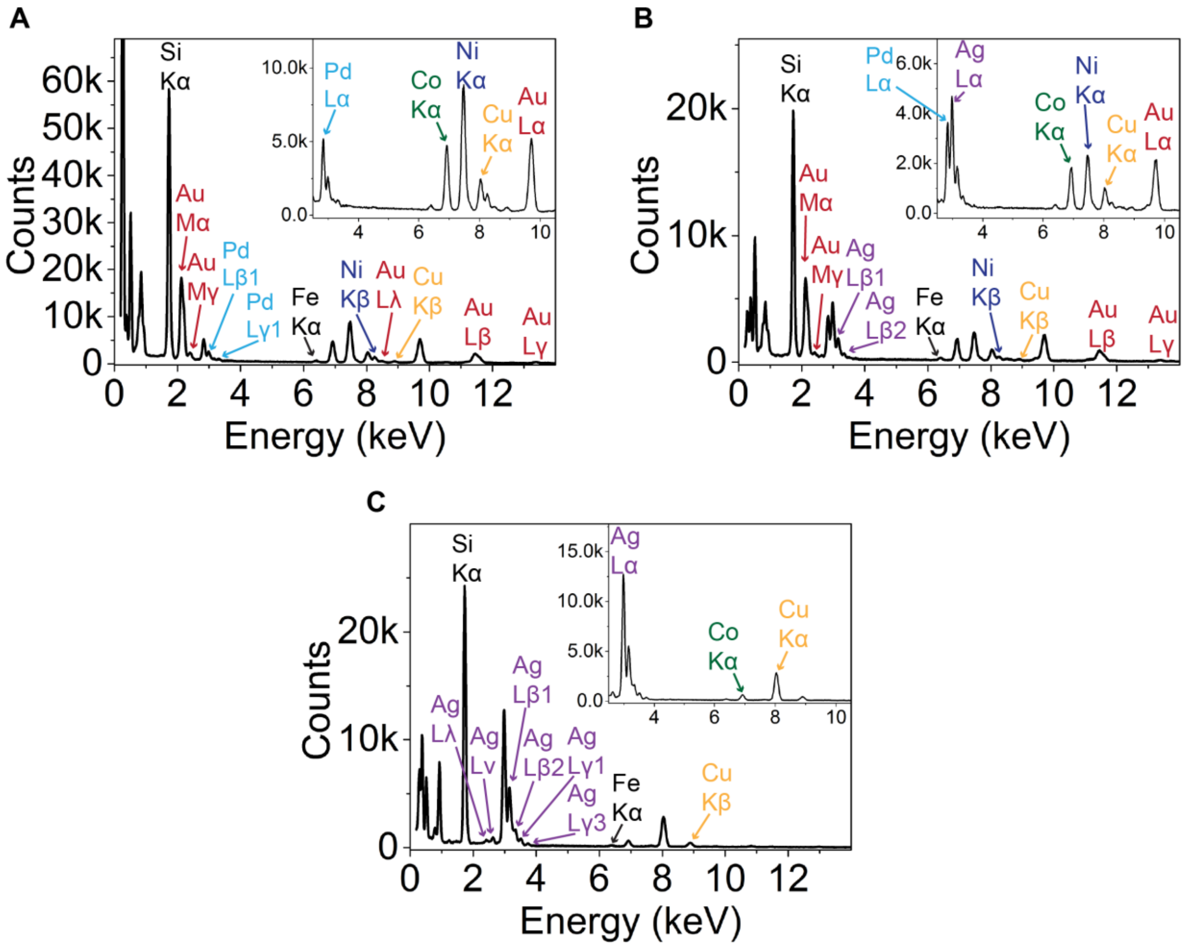

Fig. S9.

EDS spectra of the SINPs shown in Fig. 3. (A) the quinary $Au_{20}Cu_{10}Co_{20}Ni_{40}Pd_{10}$ SINP in Fig. 3A, (B) the senary $Au_{10}Ag_{10}Cu_{10}Co_{20}Ni_{40}Pd_{10}$ SINP in Fig.3B, and (C) the ternary two-interface $Ag_{30}Cu_{50}Co_{20}$ NP in Fig. 3C. Insets are enlarged regions of interest showing the peaks used for quantification. The Pd and Ag Lβ and Lγ lines in (B) overlap such that they cannot be distinguished, and labels correspond to the line with the largest contribution to each peak. Unlabeled peaks in the 0-1 keV region: C Kα (277 eV), N Kα (392 eV), O Kα (525 eV), Co Lα (776 eV), Ni Lα (851 eV, in A,B), and Cu Lα (930 eV).

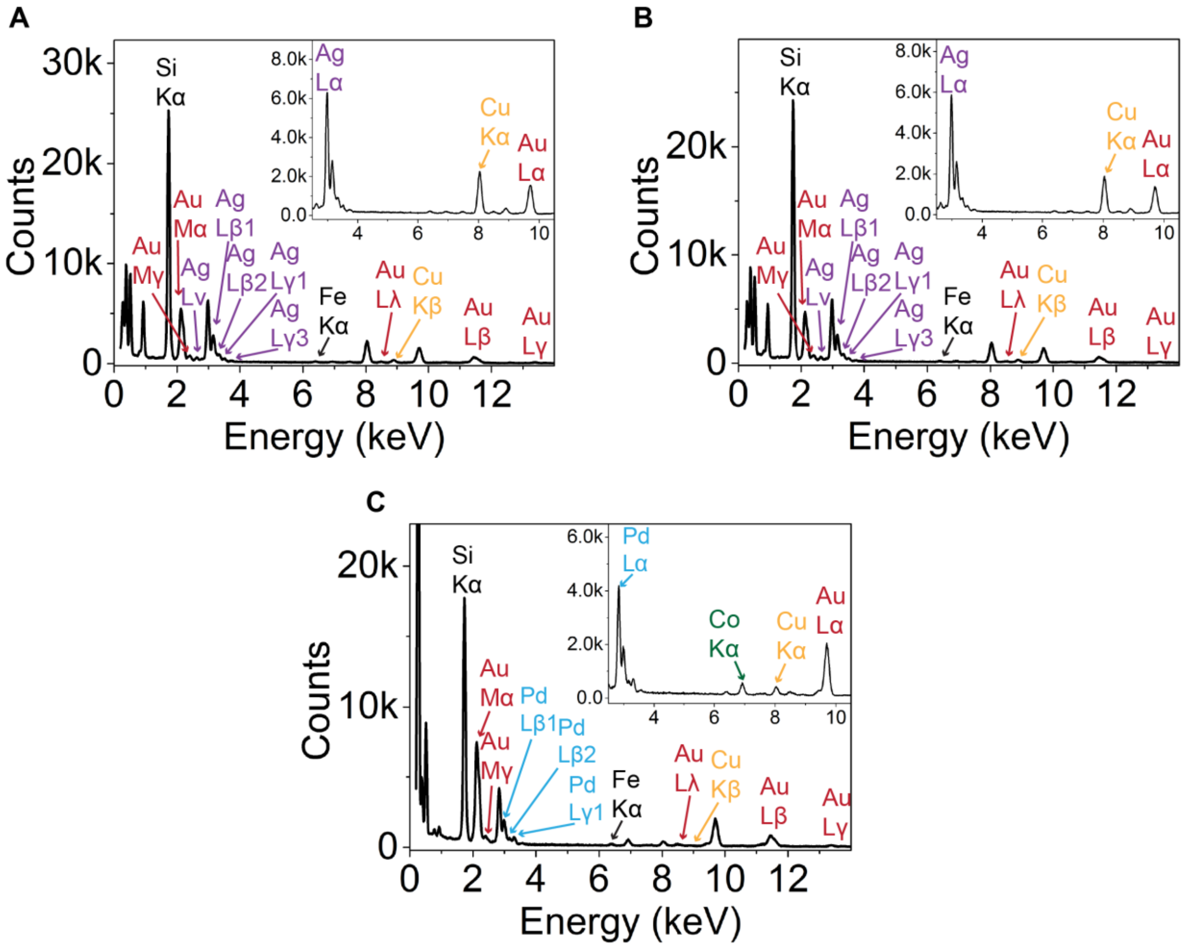

Fig. S10.

EDS spectra of the SINPs in Fig. 5. (A,B) AuAgCu, and (C) AuCuCoPd. Insets are enlarged regions of interest showing the peaks used for quantification. Unlabeled peaks in the 0-1 keV region: C Kα (277 eV), N Kα (392 eV), O Kα (525 eV), Co Lα (776 eV, in C), and Cu Lα (930 eV).

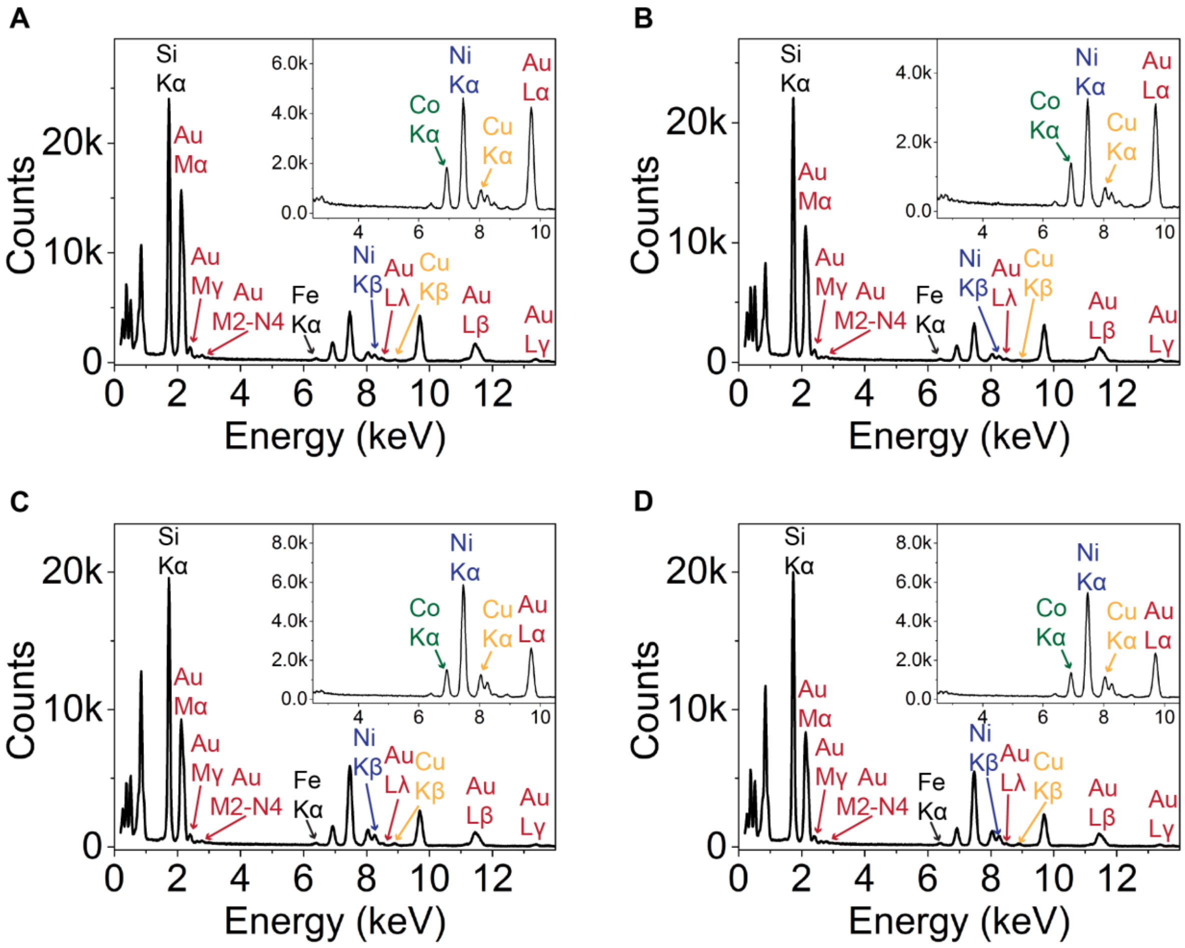

Fig. S11.

EDS spectra of the quaternary AuCuCoNi SINPs in fig. S3 after 12 h annealing. (A-D) Spectra corresponding to SINPs in fig. S3B, C, E, F, respectively. Insets are enlarged regions of interest showing the peaks used for quantification. Unlabeled peaks in the 0-1 keV region: C Kα (277 eV), N Kα (392 eV), O Kα (525 eV), Co Lα (776 eV), Ni Lα (851 eV), and Cu Lα (930 eV).

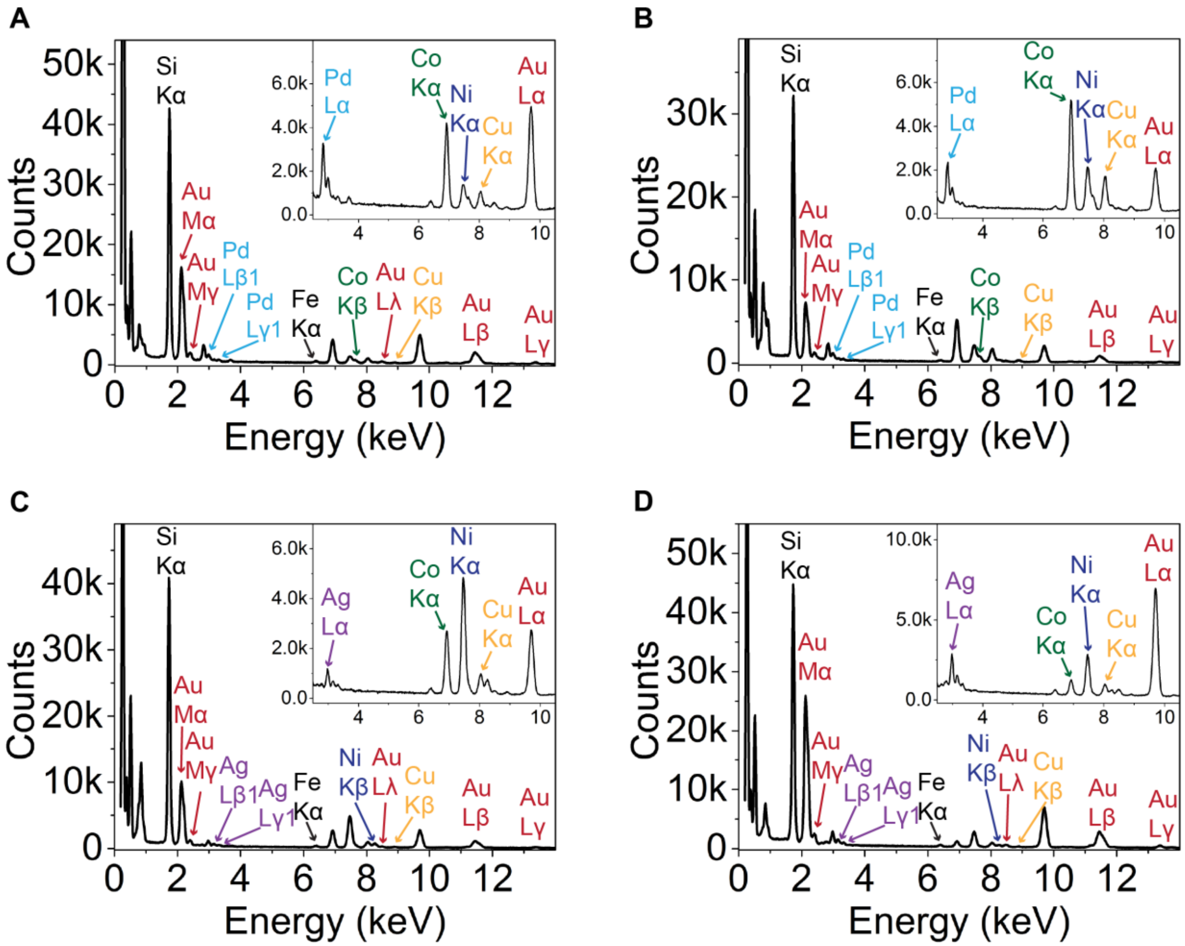

Fig. S12.

EDS spectra of the quinary SINPs in fig. S4. (A) $Au_{30}Cu_{10}Co_{40}Ni_{10}Pd_{10}$, (B) $Au_{30}Cu_{10}Co_{30}Ni_{10}Pd_{20}$, (C) $Au_{20}Ag_{10}Cu_{10}Co_{20}Ni_{40}$, and (D) $Au_{30}Ag_{10}Cu_{10}Co_{10}Ni_{40}$. Insets are enlarged regions of interest showing the peaks used for quantification. Unlabeled peaks in the 0-1 keV region: C Kα (277 eV), N Kα (392 eV), O Kα (525 eV), Co Lα (776 eV), Ni Lα (851 eV), and Cu Lα (930 eV).

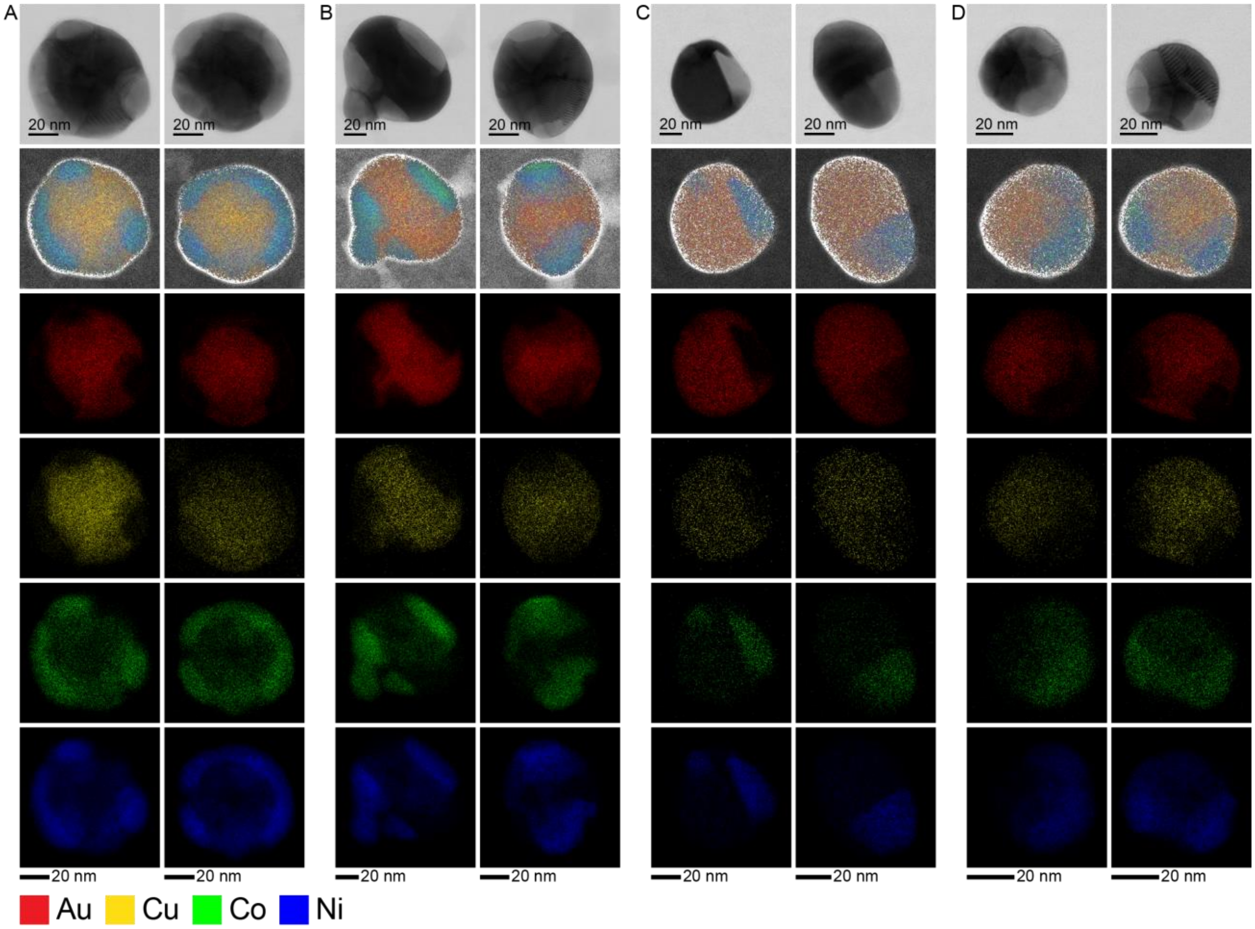

Fig. S13.

STEM-EDS of additional SINPs used for quantification in Fig. 32A. (A) $Au_{30}Cu_{20}Co_{10}Ni_{40}$. (B) $Au_{30}Cu_{10}Co_{20}Ni_{40}$. (C) $Au_{40}Cu_{10}Co_{10}Ni_{40}$. (D) $Au_{30}Cu_{10}Co_{10}Ni_{50}$.

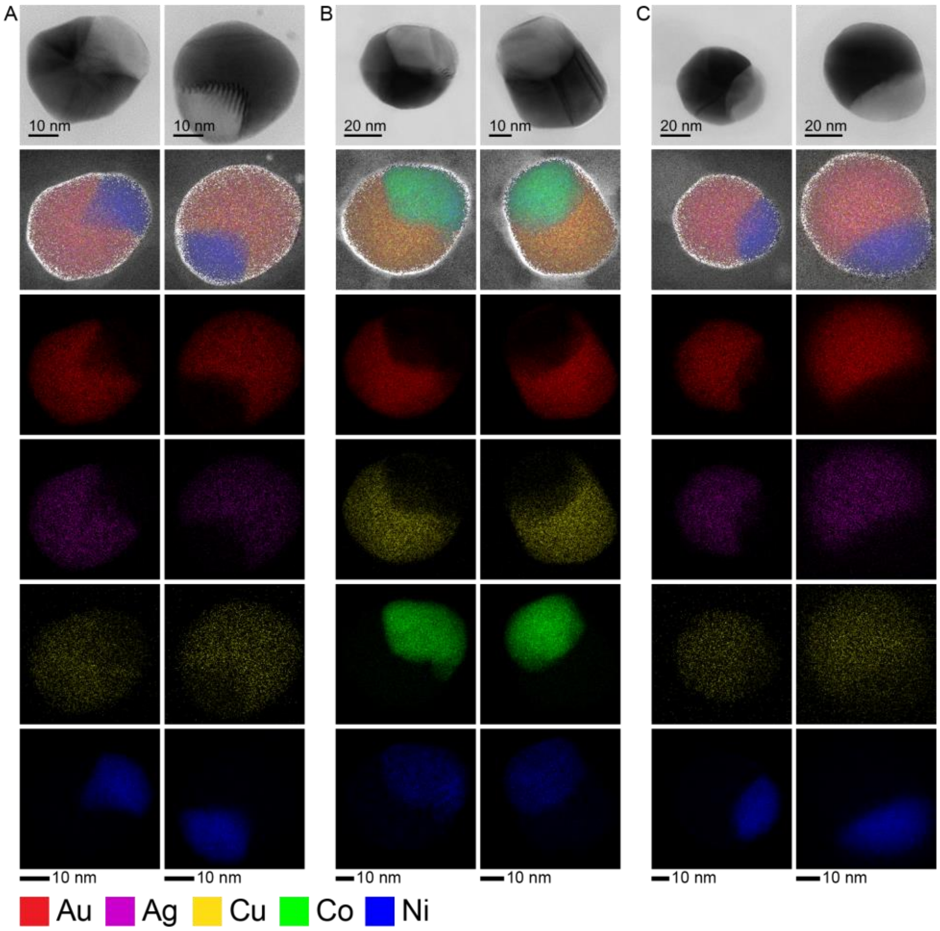

Fig. S14.

STEM-EDS of additional SINPs used for quantification in Fig. 2B. (A) $Au_{20}Ag_{10}Cu_{10}Ni_{60}$. (B) $Au_{30}Cu_{10}Co_{40}Ni_{10}$. (C) $Au_{40}Ag_{10}Cu_{10}Ni_{40}$.

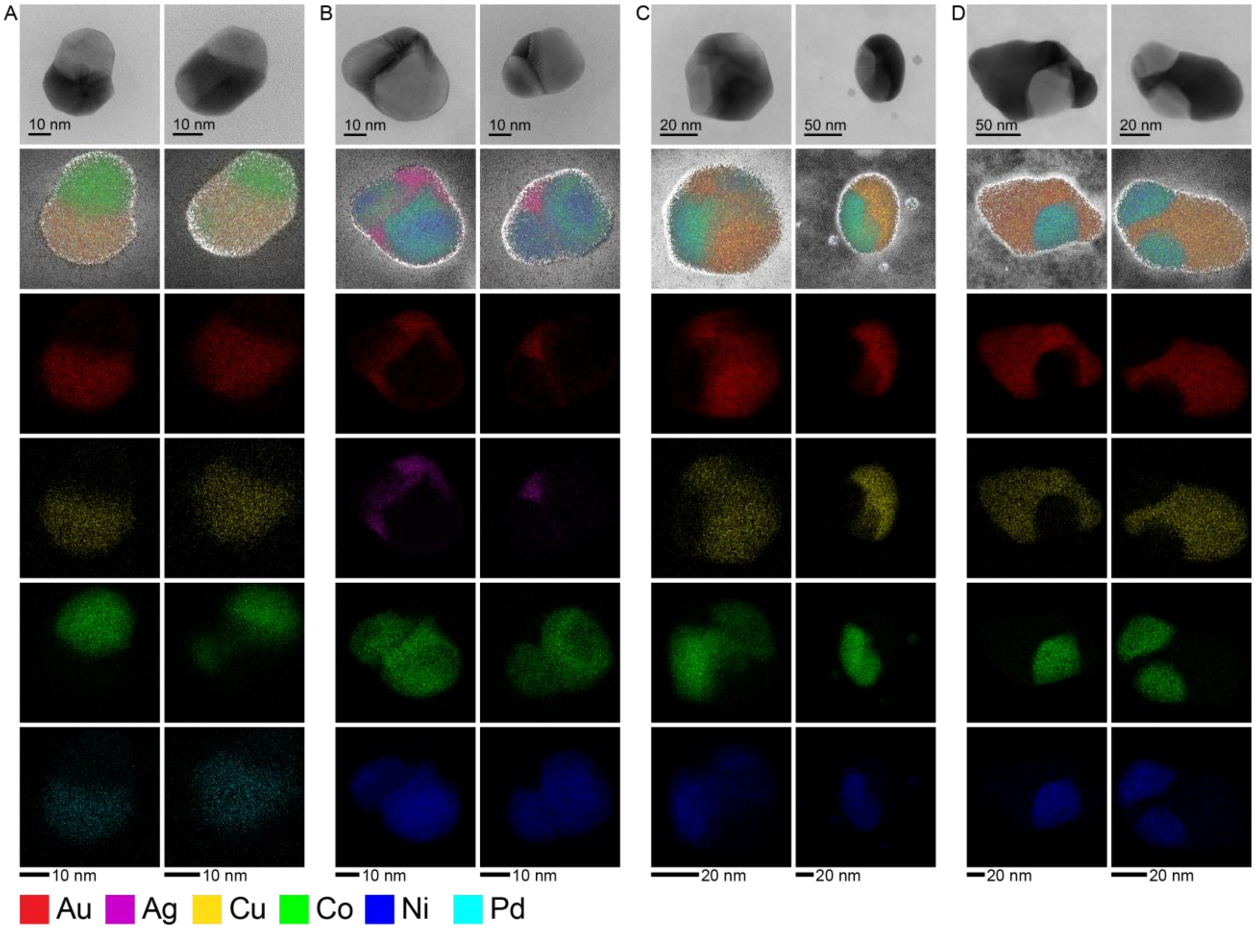

Fig. S15.

STEM-EDS of additional SINPs used for quantification in Fig. 2C. (A) $Au_{40}Cu_{10}Co_{40}Pd_{10}$. (B) $Au_{30}Ag_{10}Co_{20}Ni_{40}$. (C) $Au_{30}Cu_{20}Co_{30}Ni_{20}$. (D) $Au_{30}Cu_{20}Co_{20}Ni_{30}$.

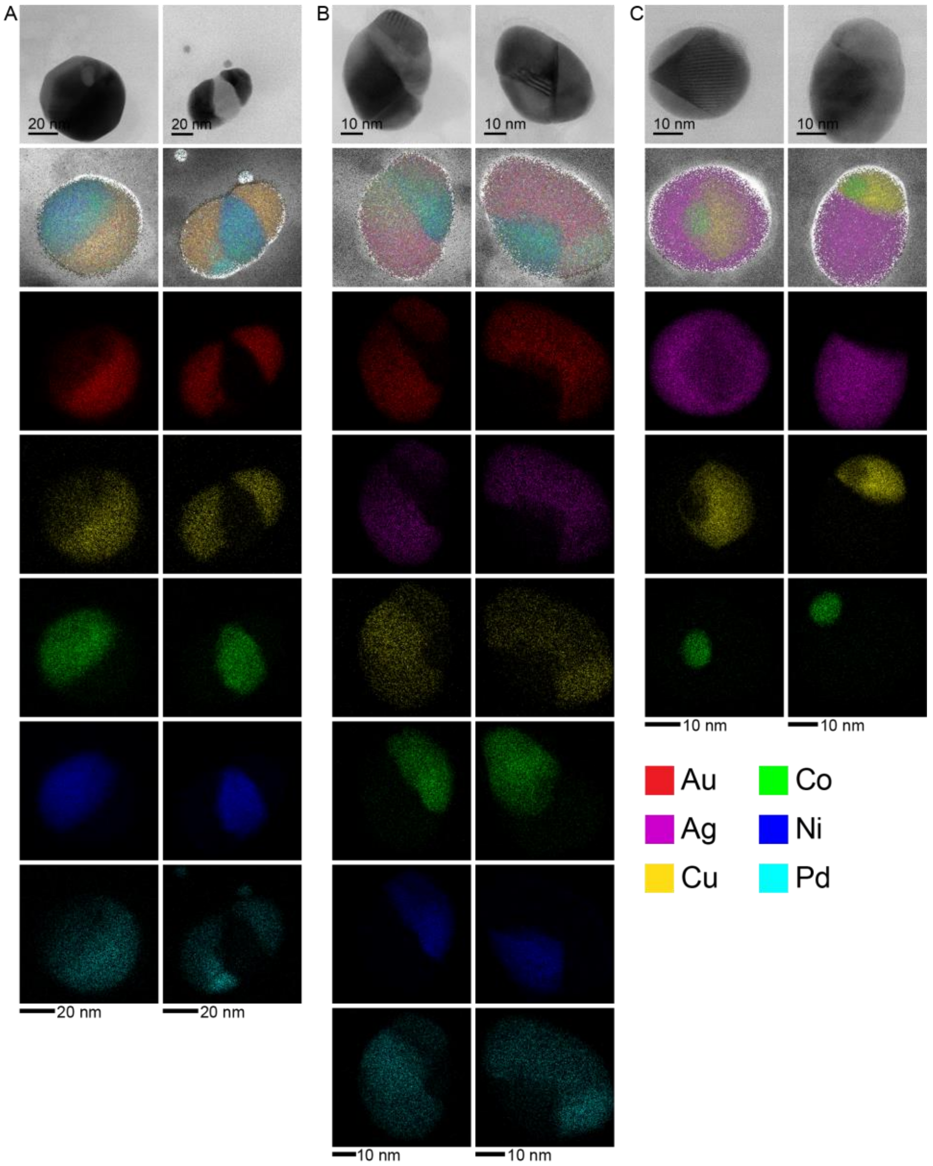

Fig. S16.

STEM-EDS of additional SINPs used for quantification in Fig 3. (A) $Au_{20}Cu_{10}Co_{20}Ni_{40}Pd_{10}$. (B) $Au_{10}Ag_{10}Cu_{10}Co_{20}Ni_{40}Pd_{10}$. (C) $Ag_{30}Cu_{50}Co_{20}$.
